# Dual-Unit ECG Acquisition System Without Shared Ground: Design and Implementation

**DOI:** 10.3390/s26196039

**Published:** 2026-09-23

**Authors:** Anna Marcucci, Enrico Baldi, Marco Grassi, Giovanni Magenes, Anna Vizziello

**Affiliations:** 1 Department of Electrical, Computer and Biomedical Engineering, University of Pavia, 27100 Pavia, Italy; marco.grassi@unipv.it (M.G.); giovanni.magenes@unipv.it (G.M.); anna.vizziello@unipv.it (A.V.); 2 S.C. Cardiologia Fondazione I.R.C.C.S. Policlinico San Matteo, 27100 Pavia, Italy; e.baldi@smatteo.pv.it; 3 Cardiac Arrest and Resuscitation Research Team (RESTART), Fondazione IRCCS Policlinico San Matteo, 27100 Pavia, Italy; 4 Consorzio Nazionale Interuniversitario per le Telecomunicazioni—CNIT, 43124 Parma, Italy

**Keywords:** electrocardiogram (ECG), wireless transmission, biomedical signal processing, MATLAB/Simulink simulation, remote health monitoring, hardware prototyping, signal acquisition, telemedicine systems

## Abstract

This paper presents the design, simulation, and experimental validation of a distributed wireless electrocardiographic (ECG) acquisition architecture based on two electrically independent sensing units and a receiving unit. The two sensing units can be positioned at different locations on the body, where they independently acquire biopotential signals and transmit them wirelessly to a common receiver. The received signals are then differentially processed to reconstruct the ECG signal without requiring a shared ground reference between the acquisition units. The receiving unit could be implemented in future wearable realizations as a portable processing device, such as a smartphone, potentially enabling additional real-time ECG analysis. The aim is to enable signal acquisition and wireless communication without a shared ground reference between devices, thereby improving user mobility and supporting wearable biomedical applications. The MATLAB/Simulink R2024b model was employed to simulate the complete sensing and communication chain, including signal acquisition, transmission, reception, and differential reconstruction. The proposed acquisition architecture was subsequently implemented on a breadboard using discrete components, with isolation amplifiers used to emulate the electrical independence of wireless transmission. In addition, a separate preliminary experiment using independent ADALM2000 platforms was conducted to investigate the feasibility of signal transmission through a short-range wireless link. Simulation results showed that conventional amplitude modulation (AM) and double-sideband suppressed-carrier (DSB-SC) transmission achieved SNRs of 20.5 dB and 28.9 dB, and RMSEs of $2.12\times 10^{-4}$ and $8.01\times 10^{-5}$, respectively, with the DSB-SC performance obtained assuming perfectly synchronized carriers at the transmitter and receiver. Experimental validation of the proposed acquisition architecture using real ECG recordings demonstrated reliable ECG reconstruction, yielding SNR values of up to 19.7 dB together with accurate heart-rate (HR) estimation. A separate preliminary experiment using two independent ADALM2000 platforms further demonstrated the feasibility of ECG signal transmission and reconstruction through a short-range wireless link, achieving a reconstruction SNR of up to 13.5 dB.

## 1. Introduction

In recent years, technological advances have significantly impacted healthcare, promoting the development of digital solutions for continuous and remote health monitoring. Among these, wearable devices have assumed a central role in the prevention and management of chronic diseases, enabling long-term physiological data acquisition outside traditional clinical environments [1]. In this context, electrocardiographic (ECG) monitoring is particularly relevant, as it allows non-invasive detection of cardiac rhythm abnormalities in ambulatory settings [2], supporting both early diagnosis and follow-up of cardiac patients.

Wireless, non-invasive, and portable cardiac monitoring systems are increasingly being investigated for effectively tracking heart activity. Traditional systems have several limitations, including the need to place electrodes on precise anatomical points [3,4] or the requirement of a right-leg driven circuit [5], which compromise practicality and user comfort in wearable scenarios. To overcome these constraints, portable and wireless solutions are being extensively investigated [6,7,8,9,10]. Devices such as adhesive patches, smart textiles, and consumer-grade wearables (e.g., smartwatches) have been developed to enhance usability and enable long-term monitoring. Although smart patches incorporating dry electrodes reduce the need for conductive gels [11], wearable ECG systems may still face challenges related to motion artifacts, skin–electrode impedance variations, and contact instability [12,13]. Furthermore, consumer devices based on optical photoplethysmography (PPG) or single-lead ECG have been reported to exhibit limitations in diagnostic accuracy during exercise or prolonged operation compared with medical-grade systems [14]. Beyond these well-known signal-quality considerations, conventional ECG systems typically rely on a shared electrical reference or common grounding strategy to ensure proper differential measurement and common-mode stabilization. While this approach is effective in traditional configurations based on a single acquisition unit, it imposes architectural constraints that may limit the flexibility of device placement on the body and reduce the modularity of wearable implementations, especially when multiple sensing modules are designed to operate as independent units. In clinical systems such as Holter monitors, this shared reference is typically maintained through wired connections between electrodes and the acquisition unit, requiring cables routed across the body. Conversely, consumer wearable devices such as smartwatches generally avoid such wiring but often rely on user interaction to close the measurement loop, for example by requiring the subject to touch an additional electrode with the opposite hand to acquire an ECG trace [15].

To address the architectural constraints discussed above, this work proposes and experimentally validates a dual-transmitter architecture in which the two acquisition units operate without a shared ground reference. A preliminary antenna-based experiment was also conducted to demonstrate the feasibility of extending the proposed architecture to wireless ECG transmission. The architecture comprises two independent acquisition and transmission nodes that operate without a shared ground reference and transmit their signals to an electrically isolated receiver. Leveraging the approach introduced in [16,17], where sensing patches are placed on the chest, the ECG waveform is reconstructed through differential processing of the independently received signals, enabling operation without a shared electrical ground reference or direct wired connection between the acquisition modules, while allowing flexible placement of the sensing units on the body.

In this work, we investigate this flexibility by evaluating sensor configurations involving both chest and wrist placements, with the aim of investigating wearable-friendly positioning of the sensing units. In particular, the investigation of wrist-based configurations explores the potential compatibility of the architecture with wrist-worn devices, such as smartwatches. Furthermore, a complete MATLAB/Simulink R2024b model was developed, covering analog amplification, filtering, transmission and differential reconstruction. The transmission stage was quantitatively analyzed by comparing conventional Amplitude Modulation (AM) and Double-Sideband Suppressed-Carrier (DSB-SC) modulation in terms of Signal-to-Noise Ratio (SNR) and Root Mean Squared Error (RMSE), providing insight into design trade-offs for wearable applications.

The proposed architecture was subsequently implemented as a discrete-component prototype, where independent analog front-ends emulate separate wireless acquisition channels, enabling validation of the acquisition architecture independently of any specific wireless implementations. Experimental validation was then carried out to assess ECG acquisition performance under different electrode configurations through SNR, heart-rate (HR) estimation, and R-peak amplitude analysis. In addition, a preliminary proof-of-concept experiment based on two independent ADALM2000 platforms and a short-range wireless link was conducted to investigate the feasibility of DSB-SC transmission and coherent signal reconstruction of the ECG signal.

The main contributions of this work can be summarized as follows:

- Design of a distributed ECG acquisition architecture based on two electrically independent sensing and transmission nodes operating without a shared ground reference, enabling flexible placement of the sensing units and supporting future wearable implementations with centralized processing.
- Development of a complete MATLAB/Simulink model including acquisition, analog front-end, modulation, transmission, reception, and differential reconstruction for ECG acquisition.
- Implementation and experimental validation of a discrete-component prototype with two electrically independent sensing units and emulated wireless links, evaluated on healthy volunteers under different electrode configurations.
- Preliminary experimental investigation of short-range wireless signal transmission and coherent reconstruction of ECG signals using two independent ADALM2000 platforms.

The remainder of this paper is organized as follows: Section 2 describes the proposed system architecture. Section 3 presents the MATLAB/Simulink simulations and quantitative performance evaluation. Section 4 describes the hardware implementation and experimental validation, including ECG acquisition under different electrode configurations and a preliminary antenna-based transmission experiment. Finally discussion and conclusions are drawn in Section 5.

## 2. System Architecture

### 2.1. Conventional ECG Reference Scheme

Conventional ECG acquisition systems rely on a shared electrical reference, typically implemented through a right-leg electrode connected to the ground of the acquisition circuitry. The ECG waveform is obtained by measuring potential differences between electrodes placed at specific anatomical locations, using a common reference to stabilize the common-mode voltage and improve measurement accuracy [18].

While effective, this configuration requires a shared ground reference between sensing elements and processing electronics, which imposes architectural constraints in distributed or modular wearable systems.

This architectural dependency motivates the development of distributed acquisition systems capable of preserving differential ECG reconstruction while eliminating the need for a common electrical reference.

### 2.2. Proposed Architecture

The proposed system, shown in Figure 1, eliminates the traditional shared reference electrode and adopts a distributed acquisition architecture based on two electrically independent sensing nodes [16,17].

Each acquisition/transmission unit (Units 1 and 2) measures a local potential difference with respect to its own reference and operates with an independent ground and power supply. The two nodes therefore function without any shared electrical connection. Specifically, as illustrated in Figure 2, Units 1 and 2 consists of a measuring electrode (*E*) and a local reference electrode (COM):

- Unit 1 comprises electrodes $E_{1}$ and $COM_{1}$;
- Unit 2 comprises electrodes $E_{2}$ and $COM_{2}$.

These two electrode pairs can be positioned on different parts of the body. In the configuration of Figure 2, the wrist was selected as a representative location because it is commonly used in wearable devices such as smartwatches, suggesting a possible integration of the proposed unit into consumer wearable platforms.

The two measured signals are transmitted independently to the receiving unit (Unit 3), where they are demodulated and processed for differential reconstruction. No shared ground connection exists between the acquisition nodes and the receiver. Consequently, the complete sensing and communication chain operates without any shared electrical reference between the acquisition nodes and the receiver.

### 2.3. Simulation and Hardware Realization

The proposed architecture was investigated through both system-level simulation and experimental implementation. In the simulation environment, the complete signal acquisition, transmission, and reconstruction chain was modeled, including the analog front-end, modulation and demodulation stages, and differential signal reconstruction. The experimental implementation reproduced the proposed acquisition and signal-processing architecture using discrete analog components, while the transmission stage was investigated through both isolated and antenna-based configurations, as described in Section 4.3.

The MATLAB/Simulink simulation model implements two independent acquisition paths corresponding to the two bio-potential signals required for differential ECG reconstruction. Each input signal is first processed by an analog front-end and subsequently applied to an independent modulation and transmission chain. Two analog modulation schemes were considered: conventional Amplitude Modulation (AM) with envelope detection at the receiver and Double-Sideband Suppressed-Carrier (DSB-SC) modulation with coherent detection. The demodulated signals are then differentially processed to reconstruct the ECG waveform. For the DSB-SC simulations, ideal carrier synchronization between the transmitter and receiver was assumed, whereas conventional AM with envelope detection does not require carrier phase synchronization.

The hardware implementation was subsequently developed using discrete analog components. At the transmitter side, TL082 operational amplifiers (Texas Instruments, Dallas, TX, USA) were used to implement a high-input-impedance buffer stage, followed by a non-inverting amplification stage with a gain of approximately 10. The high-input-impedance minimizes signal attenuation due to the skin–electrode interface impedance, which is particularly relevant in biopotential acquisition [19].

The wireless link was initially emulated using ISO122 isolation amplifiers (Texas Instruments, Dallas, TX, USA) to preserve electrical independence between the nodes, while antenna-based transmission was subsequently investigated using independent ADALM2000 platforms. Unlike a digitally sampled system, the prototype operates in the analog continuous-time domain without independent sampling clocks and therefore requires no explicit clock synchronization between the two signal paths. Under the considered short-range transmission conditions, propagation-delay mismatches are expected to be on the order of μs [20], well below the ms time scale relevant to ECG reconstruction.

At the receiver side, signal reconstruction was performed using an AD620 instrumentation amplifier (Analog Devices, Wilmington, MA, USA) configured with a gain of 2 to recover the differential ECG signal. The amplifier provides a common-mode rejection ratio (CMRR) of nearly 100 dB [21], ensuring effective suppression of common-mode interference through high-CMRR differential acquisition [22]. The device was selected for its gain programmability through the external resistor $R_{g}$, as well as its high CMRR, low input noise, and suitability for low-amplitude biopotential signal acquisition, making it particularly appropriate for ECG measurements [23].

The final signal conditioning stage was implemented using a TL084 operational amplifier (Texas Instruments, Dallas, TX, USA), providing active filtering to select the ECG bandwidth (0.5–100 Hz [24]) and additional amplification (overall gain of 10). The TL084 was selected due to its wide bandwidth, availability, and suitability for implementing active filter topologies, such as Sallen–Key configurations. Moreover, the presence of multiple integrated amplifiers within a single package enables a compact realization of the required signal conditioning stages, see Figure 3.

## 3. Simulations in Matlab/Simulink

The proposed architecture was validated through numerical simulations in MATLAB/Simulink R2024b using a block-diagram modeling approach [25]. The primary objective was to verify that accurate ECG reconstruction can be achieved without a shared ground reference, even in the presence of noise, thereby approximating realistic operating conditions.

A real ECG trace extracted from the MIT-BIH Arrhythmia Database [26] was used as input to the model. The adopted ECG signal is shown in Figure 4. To increase simulation realism, independent zero-mean additive white Gaussian noise (AWGN) was introduced at the input of each acquisition channel. The noise standard deviation was set to $\sigma =10^{-4}$ V, which provides a representative noise level for assessing the robustness of the signal reconstruction process. The mitigation of motion artifacts, EMG interference, and electrode–skin impedance variations were not specifically investigated in this study and could be addressed in future implementations using dedicated techniques (e.g., adaptive filtering, motion detection based on accelerometers) [27,28,29].

### 3.1. Acquisition Block

Figure 5 shows the acquisition block for Boards 1 and 2 from the electrodes interface to the output of the TL082 operational amplifier. For each channel, the input signal was modeled as the superposition of the ECG waveform and AWGN noise, in order to emulate realistic acquisition conditions. The two ECG inputs, denoted as V_ECG_M_V and V_ECG_P_V, represent signals with opposite polarity. This polarity inversion arises from the acquisition architecture and electrode positioning [10]. The noise components (EPS1_V and EPS2_V) were independently generated for each channel and added to the corresponding ECG signal, yielding the noisy signals V_ECG_M_N and V_ECG_P_N, which are then fed to the transmission stage.

Table 1 describes blocks shown in Figure 5.

The “TL082 Board1” and “TL082 Board2” blocks in Figure 5 implement the analog front-end of the acquisition units. The TL082 operational amplifier vas modeled in Simulink using a behavioral approach based on Simscape. The manufacturer-provided SPICE subcircuit description was automatically converted into a custom Simscape component using the *subcircuit2ssc* MATLAB function. The resulting model preserves the electrical characteristics of the device and was connected according to the reference configuration reported in the datasheet. The same procedure was applied to all other integrated components used in the system.

### 3.2. Transmission and Receiving Block

To investigate the impact of different wireless transmission strategies on signal reconstruction accuracy, two modulation/demodulation schemes were considered: conventional AM with envelope detection; DSB-SC transmission.

Each ECG signal (V_ECG_P_TX, V_ECG_M_TX) modulates a sinusoidal carrier, with frequency $f_{c}$ = 10 kHz. A modulation index lower than unity was adopted to avoid overmodulation and ensure linear operation.

In the AM scheme, demodulation at the receiver (Board 3) is performed using a full-wave rectifier-based envelope detector, followed by a second-order Sallen–Key low-pass filter with a cutoff frequency of 70 Hz [30], detailed in Figure 6, designed to suppress the residual carrier component and high-frequency harmonics introduced by rectification.

For comparison purposes, a DSB-SC scheme was implemented. Coherent demodulation is performed by multiplying the received signal by a locally generated carrier synchronized in frequency and phase with the transmitter carrier, followed by a second-order low-pass filter to remove high-frequency components.

Figure 7 illustrates the ECG signal corrupted by additive white Gaussian noise (AWGN), which is used as the input to the modulation stages, together with the signals reconstructed after AM and DSB-SC demodulation.

Both modulation schemes allow ECG waveform reconstruction in the presence of AWGN. The AM-based demodulation exhibits a slight attenuation of the R-peaks and minor waveform distortion, mainly due to the non-linear nature of envelope detection and the subsequent low-pass filtering stage. In contrast, the DSB-SC scheme provides a reconstruction that more closely matches the original ECG morphology.

A quantitative performance evaluation was conducted in terms of output Signal-to-Noise Ratio (SNR) and Root Mean Squared Error (RMSE) with respect to the original noise-free ECG signal. Specifically, the SNR was computed as

(1) $$\mathrm{SNR}=10\log_{10}\left(\frac{\sum_{n=1}^{N}x^{2}\left(n\right)}{\sum_{n=1}^{N}{\left[x\left(n\right)-\hat{x}\left(n\right)\right]}^{2}}\right)$$

where x(n) represents the reference ECG signal (Figure 4) and $\hat{x}\left(n\right)$ is the reconstructed signal after transmission and demodulation (Figure 7b and Figure 7c, respectively). The RMSE was computed as

(2) $$\mathrm{RMSE}=\sqrt{\frac{1}{N}\sum_{n=1}^{N}{\left[x\left(n\right)-\hat{x}\left(n\right)\right]}^{2}}$$

where *N* is the total number of samples. As reported in Table 2, DSB-SC achieves higher reconstruction accuracy compared to AM. This improvement is expected under the assumption of perfectly synchronized carriers required for coherent demodulation. In practical implementations, carrier frequency and phase mismatches may reduce this theoretical advantage, potentially making envelope-based AM schemes more robust in low-complexity wearable applications.

### 3.3. Processing Unit

The demodulated signals are applied to the instrumentation amplifier AD620, which performs differential amplification and provides a single-ended output. The two transmission paths use identical modulation and demodulation stages. Under the considered short-range line-of-sight conditions, residual phase differences are small enough not to affect differential reconstruction, even in the absence of a shared ground reference.

The resulting signal is subsequently processed by a TL084 operational amplifier, configured to implement second-order Sallen-Key high-pass and low-pass filter stages [31]. Finally, an additional operational amplifier within the TL084 is used to provide further amplification, yielding the final conditioned ECG output.

Both the AD620 and the TL084 were modeled starting from their SPICE descriptions, consistently with the procedure adopted for the other analog components (described in Section 3.1).

## 4. Hardware Implementation and Experimental Validation

### 4.1. Hardware Implementation

Figure 8 shows the overall architecture of the ECG acquisition system implemented as a discrete-component laboratory prototype. The system consists of three hardware units with electrically isolated grounds: two analog front-end boards (Board 1 and Board 2) dedicated to conditioning and transmitting the biopotential signals acquired by two pairs of Ag/AgCl electrodes ($E_{1}$, $COM_{1}$ for Board 1 and $E_{2}$, $COM_{2}$ for Board 2), and a receiving and processing unit (Board 3).

The hardware implementation followed the design choices discussed in Section 2.3. The ECG signals acquired at the front-end stages are transmitted to the processing unit through ISO122 isolation amplifiers, which provide galvanic isolation and emulate the operating conditions of a wireless link. At the receiver side, an AD620 instrumentation amplifier performs differential recomposition of the two transmitted signals. The reconstructed ECG waveform is subsequently filtered and amplified by a TL084-based signal conditioning stage.

Since the three units operate without a shared ground reference, each board is powered by an independent dual supply consisting of two 9 V batteries. Therefore, the acquisition electronics do not share a common reference or conductive ground path, thereby preserving the architectural principle of differential reconstruction without common ground.

### 4.2. Signal Acquisition and Validation

Experimental validation was conducted on six healthy adult volunteers (aged 25–40 years). The experiments were designed to evaluate the feasibility and performance of the proposed acquisition architecture under controlled laboratory conditions. Signal measurements were carried out using a T3DSO2304 oscilloscope (Teledyne LeCroy, Chestnut Ridge, NY, USA). connected to the output of the processing unit. Figure 9 shows the complete experimental setup during a real-time ECG signal acquisition using the proposed system.

To assess the performance of the proposed architecture under different biopotential acquisition conditions, three electrode placement configurations were evaluated, as illustrated in Figure 10:

- Configuration 1, Figure 10a: both pairs of electrodes on the chest;
- Configuration 2, Figure 10b: one pair on chest and one pair on the wrist;
- Configuration 3, Figure 10c: both pairs of electrodes on the wrists.

Figure 11 shows the ECG waveforms in the three experimental configurations for Subject 1, together with a benchmark ECG signal (Figure 11a) recorded using a high-performance TMSi REFA amplifier (Twente Medical Systems International, Oldenzaal, The Netherlands) in the standard Lead I configuration [32]. For Configurations 1 and 2 (Figure 11b,c), the ECG morphology is clearly observable, with well-defined QRS complexes exhibiting waveform characteristics comparable to those of the benchmark signal. In contrast, Configuration 3 (Figure 11d) is dominated by random noise, and no distinct ECG waveform can be reliably identified. This behavior was consistently observed across all six subjects, confirming that Configuration 3 does not provide usable ECG signal.

This behavior can be explained by the electrode positioning and the corresponding lead orientation relative to the cardiac electrical axis [33]. In Configurations 1 and 2, at least one electrode pair is positioned on the chest, closer to the heart, enabling more effective acquisition of the cardiac biopotential. Minor residual noise components are still present, as commonly observed in low-amplitude biopotential measurements. In Configuration 3, both electrode pairs are located on the wrists, resulting in a substantially attenuated differential signal due to increased distance from the heart and less favorable lead alignment. As a result, the reconstructed signal amplitude approaches the noise floor of the acquisition chain, and the output is primarily characterized by random noise. Spectral analysis of the output from Configuration 3 did not reveal any distinguishable component at the mains frequency, indicating that the observed background noise is dominated by broadband random noise rather than power-line interference. This is consistent with the interpretation that the absence of a detectable signal reflects an intrinsic attenuation of the signal, rather than a common-mode rejection limitation.

To quantitatively evaluate the proposed architecture, three complementary metrics were considered, (i) peak-to-peak SNR, (ii) HR estimation accuracy, and (iii) R-peak amplitude, in order to assess signal quality, preservation of cardiac timing information, and signal strength across configurations, respectively.

An experimental SNR analysis was performed following the approach adopted in [34]. The peak-to-peak signal-to-noise ratio (PPSNR) was computed as

(3) $$\begin{array}{c} {\mathrm{PPSNR}}_{\mathrm{dB}}=20\log_{10}\left(\frac{{\mathrm{PP}}_{QRS}}{{\mathrm{PP}}_{N}}\right) \end{array}$$

where ${\mathrm{PP}}_{QRS}$ denotes the peak-to-peak amplitude of the QRS complex and ${\mathrm{PP}}_{N}$ denotes the peak-to-peak noise amplitude measured in a baseline segment between consecutive cardiac cycles. The same procedure was applied to both the benchmark ECG and the reconstructed ECG signals. Unlike the reconstruction SNR introduced in Equation (1), which requires a reference signal to compute a sample-by-sample error, the PPSNR is a self-referenced metric computed directly within a single trace and, therefore, does not require an external reference signal. For this reason, the PPSNR has been adopted in experimental cases, where a directly comparable reference signal is not available.

For each subject, the PPSNR was calculated for every R-peak detected in the trace, and the resulting values were averaged to obtain a single PPSNR per subject (reported in Table 3). The benchmark REFA signal yielded a mean PPSNR of 25.50 ± 1.94 dB across the six subjects, while the proposed architecture achieved 19.68 ± 3.11 dB in Configuration 1 and 15.34 ± 1.59 dB in Configuration 2.

These results confirm the expected degradation in signal quality as electrode placement moves farther from the chest.

In order to quantitatively support the dominance of random noise in the acquisitions from Configuration 3, the PPSNR was also calculated for that configuration using the R-peak positions identified by the signal acquired from the REFA instrument, which was used simultaneously, under the assumption of temporal correspondence. Among the six subjects, the average PPSNR was 0.14 ± 0.86 dB, indicating that the expected QRS amplitude is statistically indistinguishable from the local background noise.

The preservation of physiologically relevant temporal information was assessed through HR analysis, with HR estimated from the R–R intervals extracted from the detected R-peaks.

To further assess cardiac timing preservation, a preliminary comparison was performed among the six subjects using the proposed system and a high-performance TMSi REFA amplifier operated simultaneously. Following the comparative approach adopted in [35], the relative HR estimation error was computed as

(4) $$E_{HR}\left(\%\right)=\frac{\left|HR_{REFA}-HR_{Board}\right|}{HR_{REFA}}\times 100$$

where $HR_{REFA}$ and $HR_{Board}$ denote the heart-rate values estimated from the benchmark REFA acquisition and the proposed system, respectively. Across the six subjects, the HR estimation error remained up to 0.24% for Configuration 1 (mean 0.14%) and up to 1.16% for Configuration 2 (mean 0.52%), confirming reliable preservation of cardiac timing information across participants. Four repeated measurements were performed for each subject and configuration, with error percentages below 2%, indicating good agreement with the benchmark measurements.

Additionally, HR values estimated from the acquired ECG signals in Configurations 1 and 2 ranged from 74.9 bpm to 87.8 bpm across all subjects, further demonstrating the feasibility of cardiac cycle detection under the tested experimental conditions.

The quality of ECG waveform preservation was also evaluated by calculating the Pearson correlation coefficient (ρ) [36] between the REFA references signals and the signals acquired using the proposed system for Configuration 1 and 2. Averaging across the six subjects, the resulting correlation was 0.789 for Configuration 1 and 0.639 for Configuration 2, indicating good preservation of the overall morphology of the waveforms, with Configuration 1 showing grater fidelity, consistent with the lower HR estimation errors reported earlier.

Finally, the ECG signal strength in Configurations 1 and 2 was quantitatively evaluated using the average R-peak amplitudes and corresponding standard deviations across all six subjects, as summarized in Table 4. The amplitude of the R-peaks was selected as a representative measure of the ECG signal level because of its relevance to heartbeat detection.

The quantitative results confirm the trend already observed in the waveforms: Configuration 1 consistently provides higher ECG amplitudes, whereas Configuration 2 exhibits reduced signal levels. The larger R-peak amplitudes observed in Configuration 1 are attributed to the chest electrode pair, which provides a stronger differential ECG component. Despite these variations, QRS complexes remain clearly identifiable in both Configurations 1 and 2, whereas P and T waves are less prominent due to their lower intrinsic amplitude and residual noise.

### 4.3. Preliminary ECG Transmission Experiment

Although the prototype presented in the previous sections uses isolation amplifiers to emulate the electrical independence provided by a wireless link, an additional preliminary experiment was carried out to investigate the feasibility of ECG transmission through a short-range wireless link.

The proof-of-concept experiment was implemented using two independent ADALM2000 platforms controlled from separate PCs through MATLAB. The ADALM2000 platforms were used as programmable analog interfaces for the transmitter and receiver. One platform was configured as the transmitter, while the second operated as the receiver. The experimental setup is shown in Figure 12. The transmitting antenna was connected to the analog output of the transmitting platform, whereas the receiving antenna was connected to the analog input of the receiving platform. No wired communication link or data exchange was established between the two PCs, so that signal transfer between the transmitter and receiver relied exclusively on the wireless link.

The ECG waveform was modulated using a DSB-SC scheme and transmitted through a short-range link using two antennas. A carrier frequency of 1 MHz was selected. The experiment was performed under short-range line-of-sight conditions. The signal received by the second ADALM2000 was coherently demodulated and filtered using a fourth-order Butterworth low-pass filter with a cutoff frequency of 150 Hz to recover the ECG baseband signal.

An ECG segment comprising two consecutive cardiac cycles was selected for transmission experiment. Figure 13 shows the transmitted and the reconstructed ECG waveform in Figure 13a and Figure 13b, respectively.

The temporal offset between the transmitted and reconstructed waveforms was estimated through cross-correlation and compensated before evaluating the reconstruction performance using the Pearson correlation coefficient (ρ), reconstruction SNR, and RMSE, defined in (1) and (2). To specifically evaluate the preservation of the cardiac waveform morphology, the same metrics were averaged over the isolated QRS complexes. The complete waveform and QRS-specific results are reported in Table 5. The QRS reconstruction achieved a correlation coefficient of ρ=0.95, a reconstruction SNR of 13.53 dB and an RMSE of 0.06.

Although the overall waveform similarity is affected by baseline fluctuations and low-frequency components, the high correlation coefficient and low reconstruction error obtained for the isolated QRS complexes indicate that the transmission link preserves the main morphological features of the ECG waveform. Accordingly, the lower similarity observed over the complete waveform primarily reflects baseline and low-frequency variations rather than substantial distortion of the QRS complexes.

The objective of this experiment was to provide a preliminary proof-of-concept of actual wireless ECG transmission and reconstruction. The results provide an initial experimental demonstration of the feasibility of transmitting and reconstructing an ECG waveform through a short-range wireless link. Although no specific wireless protocol is assumed in the present work, low-power technologies such as Bluetooth Low Energy (BLE) [37] could represent a possible implementation option for future wearable realizations.

## 5. Discussion and Conclusions

This paper presented a distributed ECG acquisition architecture composed of two electrically independent acquisition units and a receiving unit, enabling differential ECG reconstruction without a shared ground reference. By separating the acquisition nodes electrically, the proposed architecture provides greater flexibility in electrode placement and a basis for modular wearable ECG systems. Previous studies ([16,17]) have been limited to placing the acquisition units on the chest and lower limbs to replicate the standard configurations of leads I, II, and III. This study extends these investigations. First, electrode placement was expanded beyond the standard configurations to evaluate the flexibility of the proposed architecture and its compatibility with wearable devices (particularly wrist-worn devices). Furthermore, a wide range of quantitative metrics was evaluated across multiple subjects, rather than relying on a single-subject or purely qualitative assessment.

The experimental results highlight the importance of electrode placement in determining the amplitude of the differential cardiac signal. Signal quality depends on the anatomical locations of the electrode pairs: the ECG waveform was clearly detectable when both electrode pairs were positioned on the chest, remained clearly identifiable with one pair on the chest and one on the wrist, although with reduced amplitude, and was not reliably detectable when both pairs were positioned on the wrists. These findings demonstrate that the proposed architecture can preserve the differential ECG information required for cardiac waveform reconstruction while allowing the two acquisition units to be positioned independently. It should be noted that these results were obtained under controlled experimental conditions; motion artifacts, EMG interference, and variations in electrode–skin impedance were not taken into account in this study and are considered a separate issue, typically addressed through specific techniques such as adaptive filtering or motion detection based on accelerometers.

Beyond electrode placement, differential reconstruction also depends on how well the two acquisition paths are aligned in time. DSB-SC coherent demodulation was simulated assuming ideal carrier synchronization, while AM modulation with envelope detection, which does not require such synchronization, was also examined. Since the prototype operates in continuous-time analog mode, synchronization between the acquisition units is not necessary; however, it is essential to keep the relative delay between the two paths negligible, which is ensured by the isolation amplifiers.

A separate preliminary experiment further investigated the feasibility of transmitting an ECG waveform through a short-range wireless link. The successful reconstruction of the transmitted waveform, together with the preservation of the main ECG morphological features, provides an initial validation of the wireless transmission and coherent demodulation stages under the tested conditions.

This test, however, did not take into account channel effects such as attenuation, noise, and interference. Future work will focus on the experimental validation of the proposed architecture under more realistic operating conditions, including practical implementations on wearable devices and a comprehensive characterization of wireless communication performance, as well as on the exploration of digital modulation schemes.

## Figures and Tables

**Figure 1:**
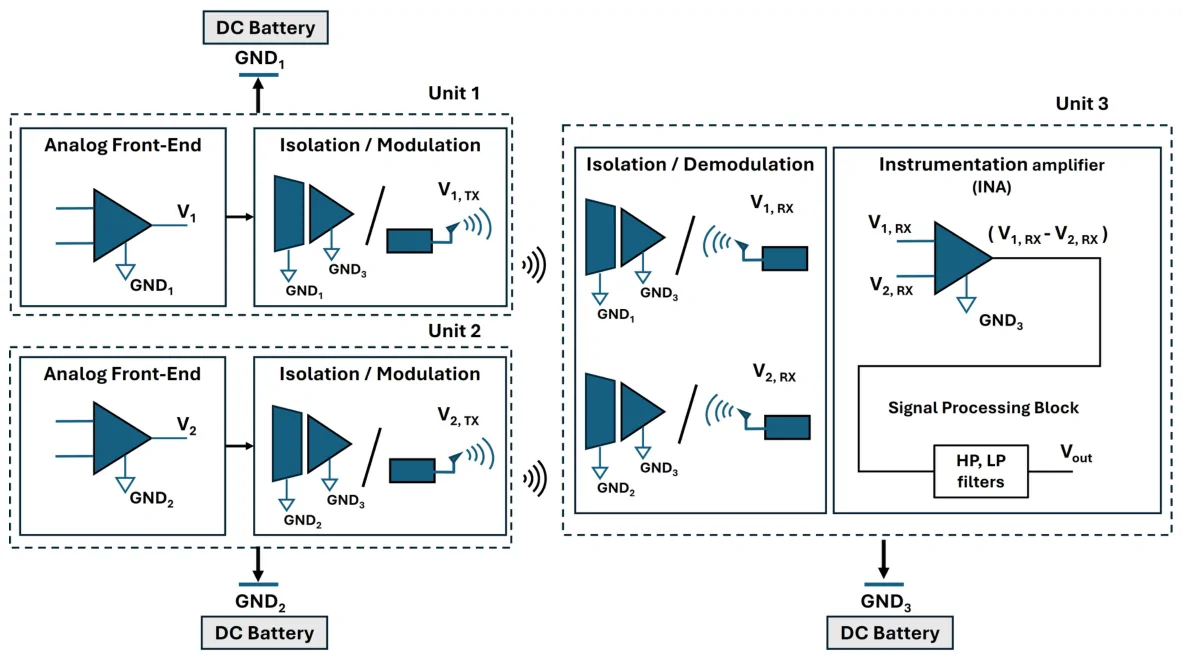
Block diagram of the dual-transmitter wireless ECG acquisition system. Two independent acquisition/transmission nodes (Unit 1 and Unit 2), each powered by a separate battery and operating without a shared ground reference ($GND_{1}$ and $GND_{2}$), acquire and transmit the ECG potentials through isolation/modulation stages. At the receiver (Unit 3), the signals are independently demodulated and processed by an instrumentation amplifier to reconstruct the differential ECG waveform ($V_{1,\mathrm{RX}}-V_{2,\mathrm{RX}}$), followed by analog high-pass (HP) and low-pass (LP) filtering. The proposed architecture ensures electrical independence among all modules ($GND_{1}$, $GND_{2}$, $GND_{3}$) and enables flexible electrode placement for wearable applications.

**Figure 2:**
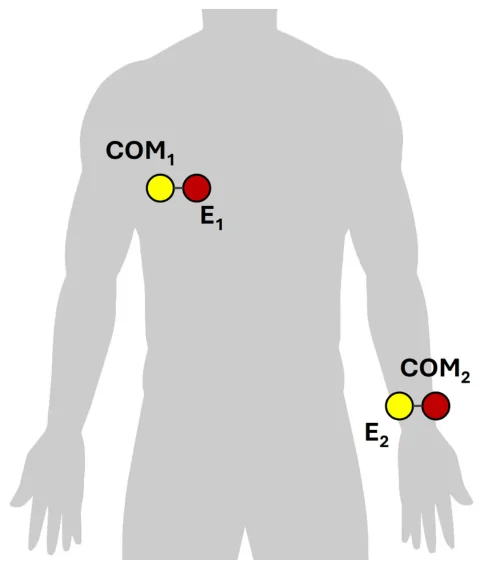
Example placement of the two independent electrode pairs used in the proposed distributed ECG acquisition system. Unit 1 (E_1_, COM_1_) is positioned on the chest, while Unit 2 (E_2_, COM_2_) is positioned on the wrist, which is a comfortable and easily accessible position for the user, promoting daily use of the device.

**Figure 3:**
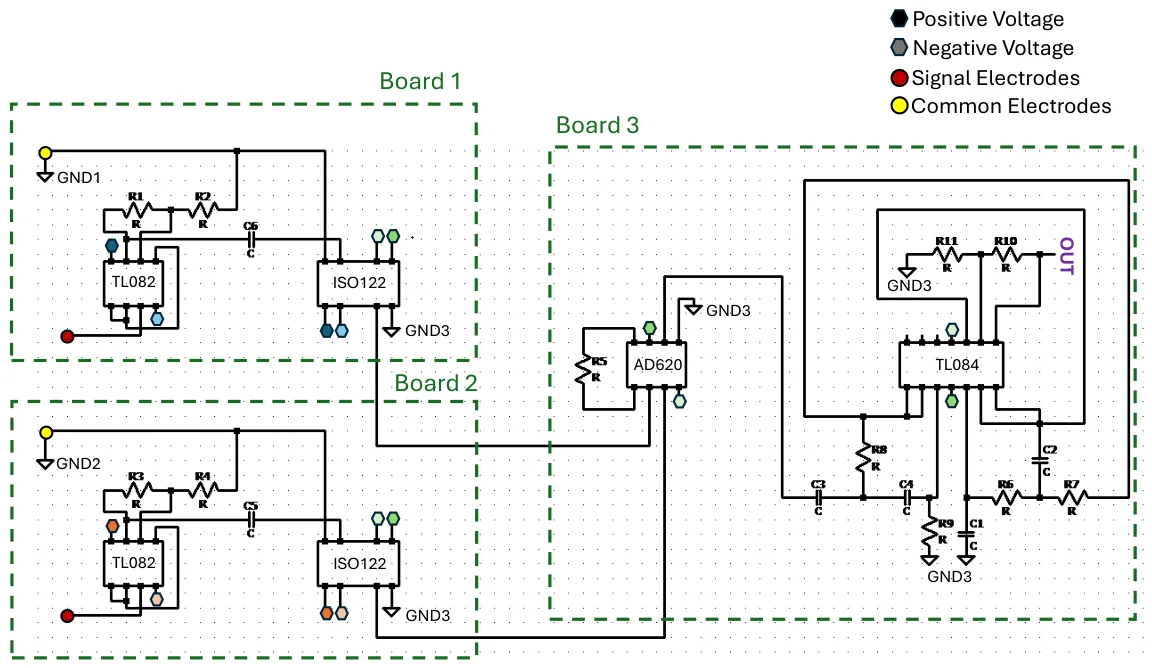
Experimental hardware implementation of the ECG acquisition system. Boards 1 and 2 correspond to the independent acquisition/transmission units, while Board 3 implements the receiving and differential reconstruction stage. The yellow circles represent the $COM_{1}$ and $COM_{2}$ electrodes, while the red circles represent the $E_{1}$ and $E_{2}$ electrodes. Electrical isolation between modules is achieved using ISO122 isolation amplifiers.

**Figure 4:**
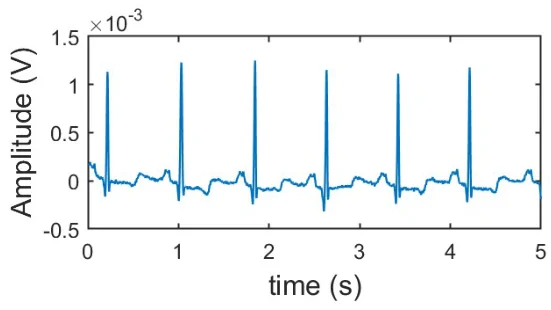
ECG trace extracted from the MIT-BIH Arrhythmia database [26], used for system simulation.

**Figure 5:**
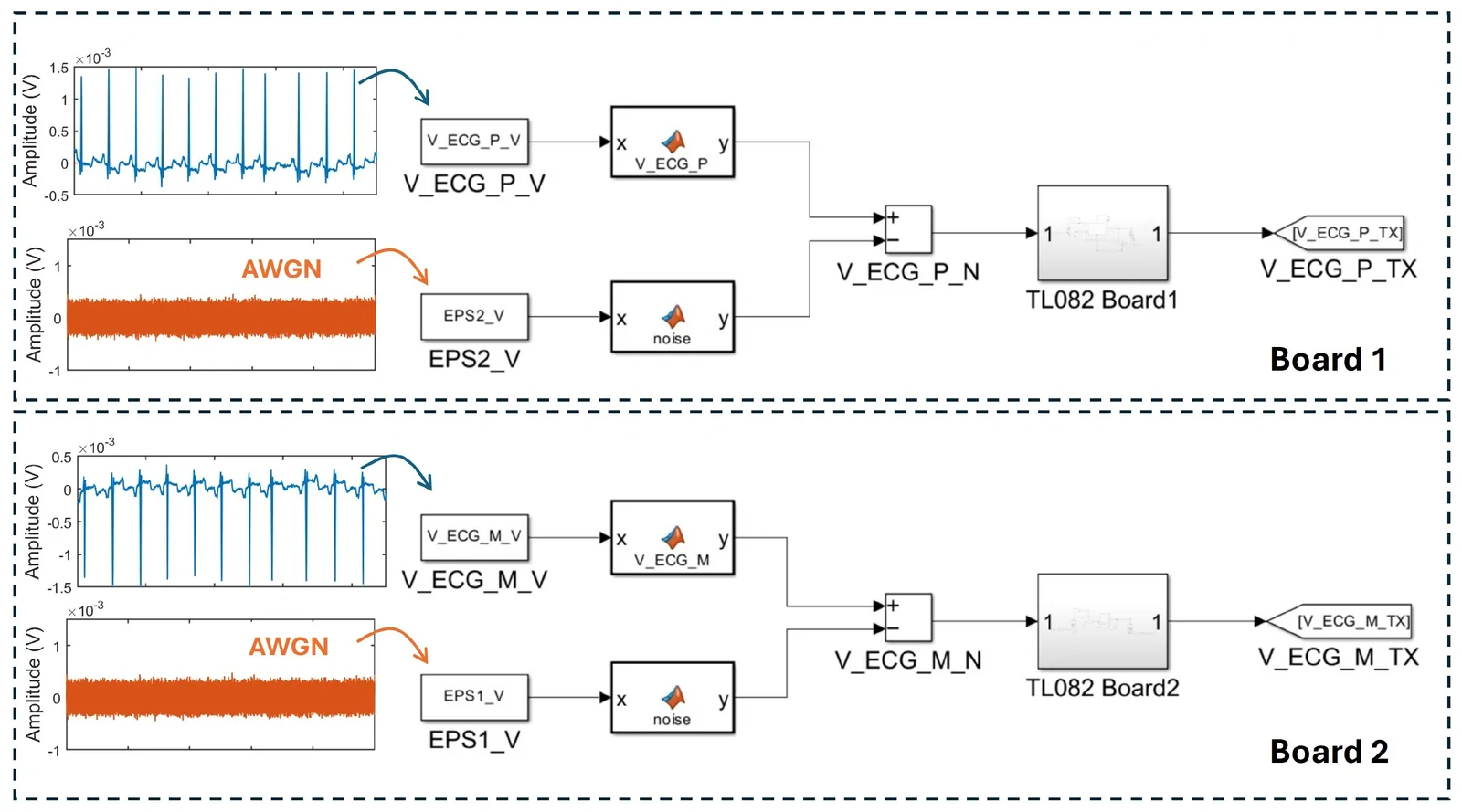
Acquisition blocks for Board 1 and Board 2, from the electrodes measurement to the TL082 chip output. The inputs (ECG signals in blue and noise in red) of channels were simulated using Simulink’s *Constant* blocks. The ECG peaks are on the order of mV, while the noise standard deviation is $\sigma =10^{-4}$ V, representative of realistic acquisition conditions. The AMP block contains the TL082 chip model.

**Figure 6:**
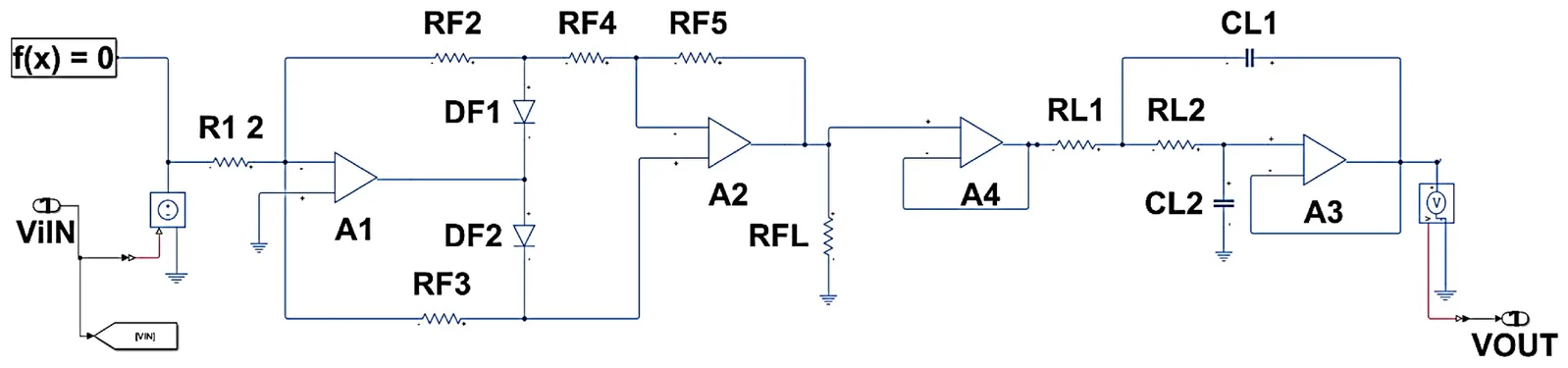
Simulink implementation of the full-wave rectifier-based envelope detector, followed by a second-order Sallen–Key low-pass filter.

**Figure 7:**
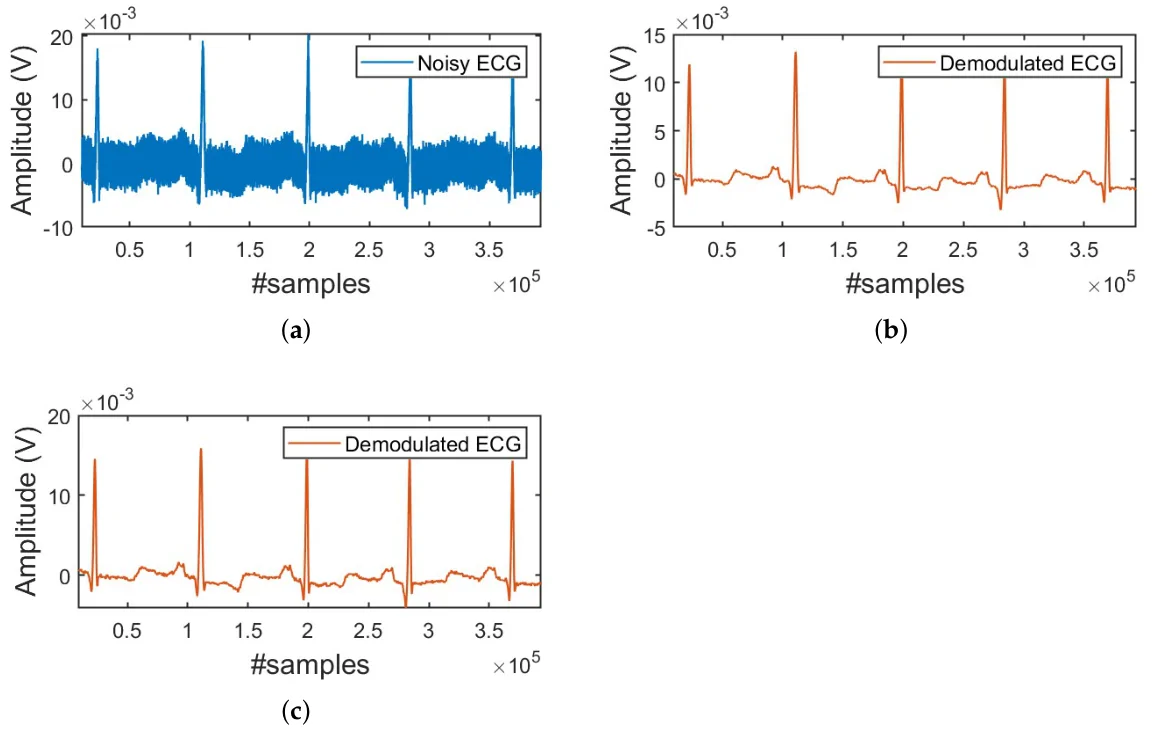
Comparison between the noisy ECG signal, used as the input to the modulation stages, and the reconstructed signals obtained after AM and DSB-SC demodulation. The input ECG in blue (**a**), is corrupted by additive white Gaussian noise ($\sigma =10^{-4}$ V). The red traces represent the demodulated signals for (**b**) AM with envelope detection and (**c**) DSB-SC with coherent detection. All signals are shown after the front-end amplification stage (gain of 10), applied prior to transmission.

**Figure 8:**
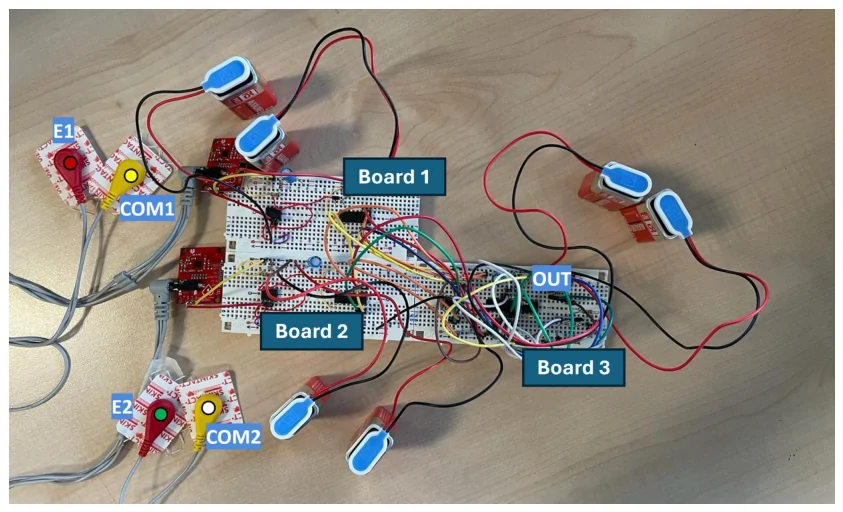
Overall architecture of the breadboard-based ECG acquisition system composed of three hardware units: two analog front-end boards for biopotential signal conditioning and transmission (Board 1 and Board 2); one board for signal reception, processing, and filtering (Board 3). Red electrodes correspond to $E_{1}$ and $E_{2}$ of Board 1 and Board 2, respectively, while yellow electrodes correspond to the $COM_{1}$ and $COM_{2}$ reference electrodes.

**Figure 9:**
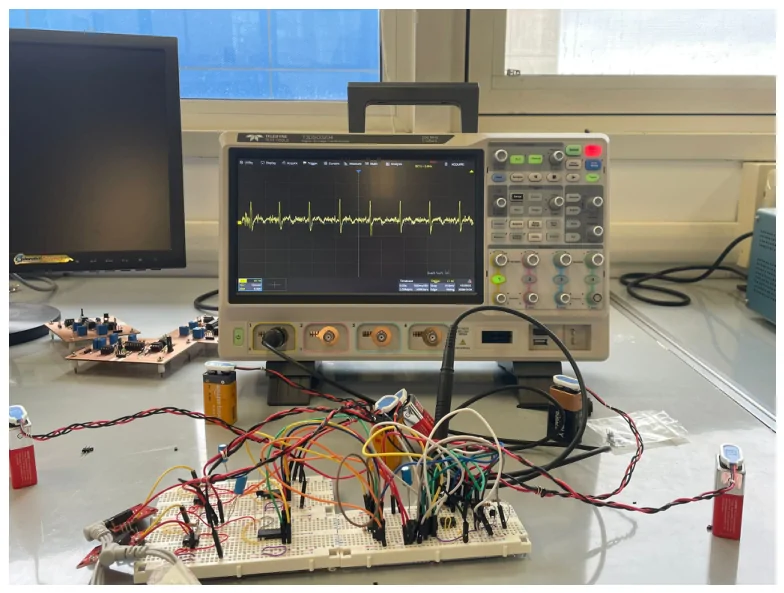
Experimental ECG signal acquired from the hardware prototype and visualized on the oscilloscope. The waveform demonstrates the qualitative reconstruction capability of the system under real operating conditions.

**Figure 10:**
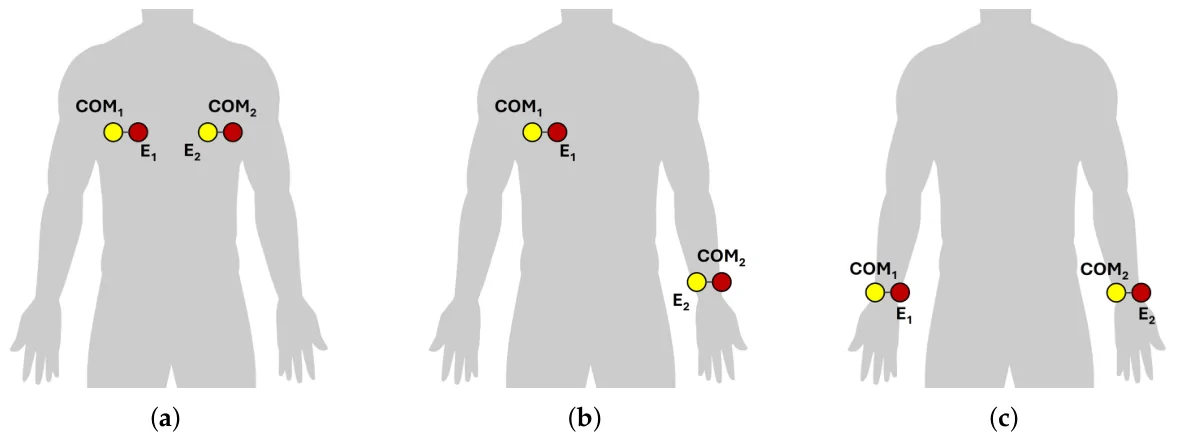
Schematics of the tested electrode placement configurations: (**a**) both pairs on the chest; (**b**) one pair on the chest and one on the wrist; (**c**) both pairs on the wrists.

**Figure 11:**
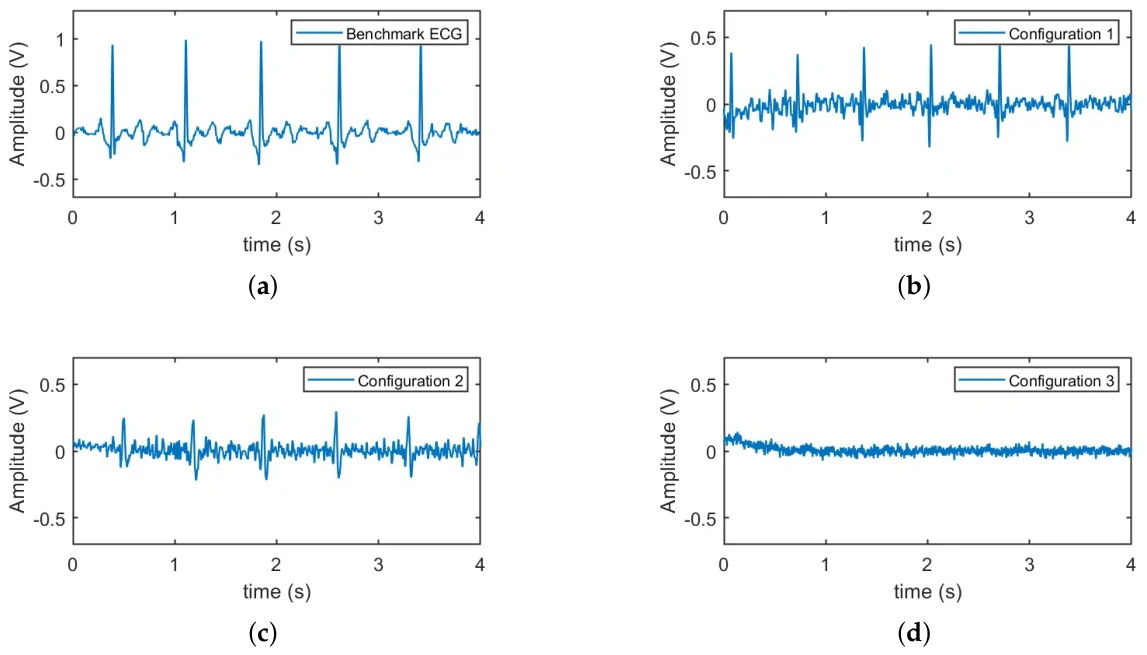
Lead-I benchmark ECG (**a**) and signals acquired with electrode pairs positioned according to the configurations shown in Figure 10: (**b**) both pairs on chest; (**c**) one pair on chest and one on the wrist; (**d**) both pairs on wrists.

**Figure 12:**
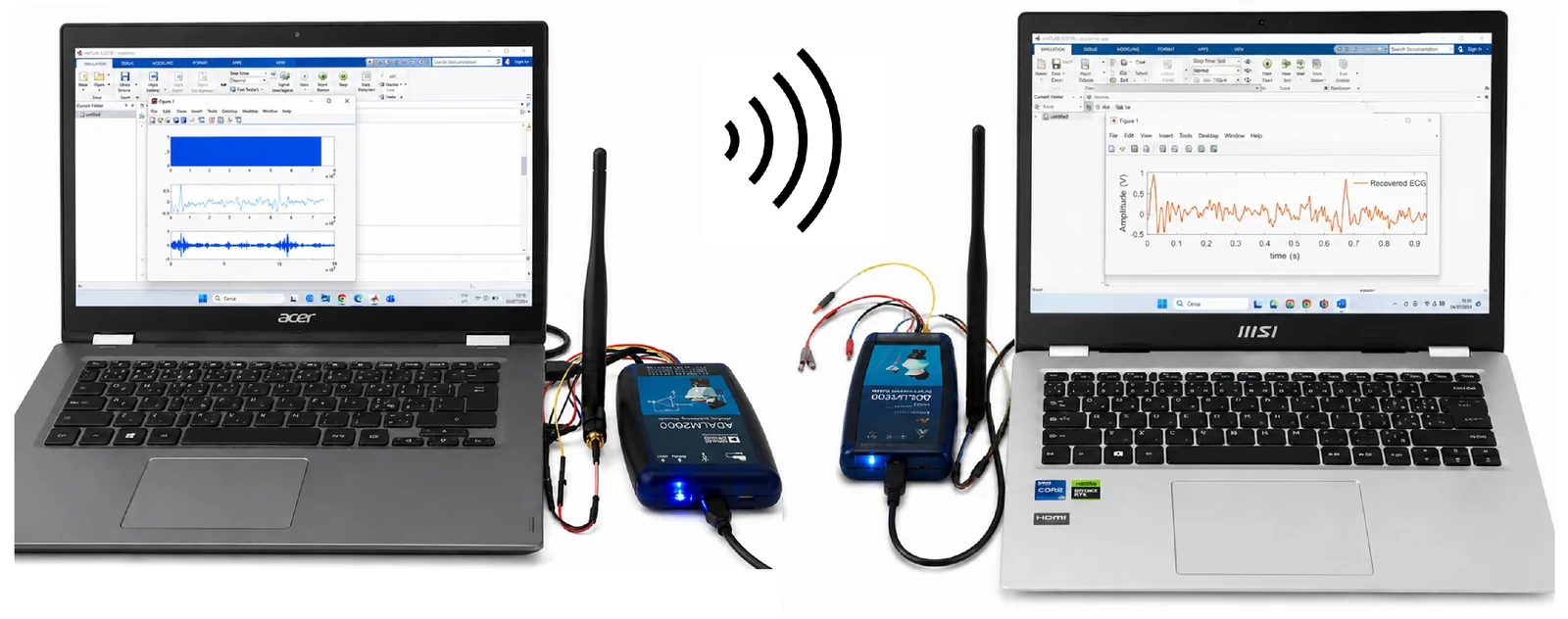
Experimental setup for the preliminary transmission of an ECG waveform through a short-range wireless link using two independent ADALM2000 platforms.

**Figure 13:**
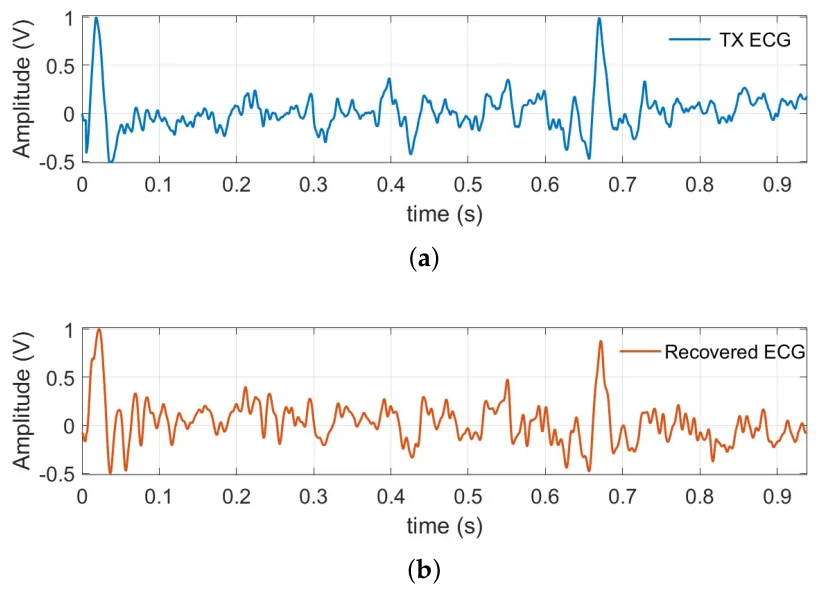
(**a**) Transmitted ECG waveform; (**b**) Reconstructed ECG waveform after reception and demodulation.

**Table 1:** Blocks in Figure 5.

Blocks	Description
V_ECG_M_V, V_ECG_P_V	ECG input signals (V)
EPS1_V, EPS2_V	AWGN components (V)
V_ECG_M_N, V_ECG_P_N	Noisy ECG signals
V_ECG_M_TX, V_ECG_P_TX	Signals at transmission stage input

**Table 2:** Performance comparison between AM and DSB-SC.

Modulation	SNR (dB)	RMSE
AM	20.47	$2.12\times 10^{-4}$
DSB-SC	28.92	$8.01\times 10^{-5}$

**Table 3:** Subject-level PPSNR (dB) for the REFA benchmark and the proposed system (Configurations 1 and 2).

Subject	Benchmark REFA	Configuration 1	Configuration 2
1	23.81	18.80	16.60
2	24.85	19.45	15.33
3	27.35	18.62	15.66
4	23.95	14.98	12.34
5	28.46	23.75	15.33
6	24.56	22.45	16.76
**Mean ± SD**	25.50 ± 1.94	19.68 ± 3.11	15.34 ± 1.59

**Table 4:** Average R-peak amplitudes and standard deviations for the six subjects and across the six subjects in Configurations 1 and 2. Values are reported as mean ± standard deviation (in mV).

Subject	Configuration 1	Configuration 2
1	0.391 ± 0.026	0.242 ± 0.030
2	0.294 ± 0.023	0.218 ± 0.019
3	0.286 ± 0.017	0.228 ± 0.024
4	0.408 ± 0.019	0.250 ± 0.021
5	0.350 ± 0.028	0.226 ± 0.018
6	0.360 ± 0.020	0.270 ± 0.043
**Mean ± SD**	0.35 ± 0.05	0.24 ± 0.02

**Table 5:** Reconstruction performance evaluated on the complete ECG waveform and isolated QRS complexes.

Signal Segment	Pearson Coefficient (ρ)	SNR (dB)	RMSE
Full ECG waveform	0.77	3.56	0.12
QRS complexes	0.95	13.53	0.06

## Data Availability

The data presented in this study are available in “Dual-Transmitter ECG Dataset” at https://doi.org/10.5281/zenodo.19253148.

## Abbreviations

The following abbreviations are used in this manuscript:

ECG	Electrocardiogram
TX	Transmitter
RX	Receiver
AM	Amplitude Modulation
DSB-SC	Double Sideband Suppressed Carrier
ISO	Isolation Amplifier
SNR	Signal-to-Noise Ratio
RMSE	Root Mean Squared Error
HR	Heart-Rate
